# Rapid and Non-Destructive Estimation of Moisture Content in Caragana Korshinskii Pellet Feed Using Hyperspectral Imaging

**DOI:** 10.3390/s23177592

**Published:** 2023-09-01

**Authors:** Zhihong Yu, Xiaochao Chen, Jianchao Zhang, Qiang Su, Ke Wang, Wenhang Liu

**Affiliations:** College of Mechanical and Electrical Engineering, Inner Mongolia Agricultural University, Hohhot 010018, China; yzhyq@imau.edu.cn (Z.Y.); chenxiaochao@emails.imau.edu.cn (X.C.); suqi0203@emails.imau.edu.cn (Q.S.); wangke1889@163.com (K.W.); lwh20222022@126.com (W.L.)

**Keywords:** hyperspectral, Caragana korshinskii pellet feed, moisture content, rapid and non-destructive estimation

## Abstract

Moisture content is an important parameter for estimating the quality of pellet feed, which is vital in nutrition, storage, and taste. The ranges of moisture content serve as an index for factors such as safe storage and nutrition stability. A rapid and non-destructive model for the measurement of moisture content in pellet feed was developed. To achieve this, 144 samples of Caragana korshinskii pellet feed from various regions in Inner Mongolia Autonomous Region underwent separate moisture content control, measurement using standard methods, and captured their images using a hyperspectral imaging (HSI) system in the spectral range of 935.5–2539 nm. The Monte Carlo cross validation (MCCV) was used to eliminate abnormal sample data from the spectral data for better model accuracy, and a global model of moisture content was built by using partial least squares regression (PLSR) with seven preprocessing techniques and two spectral feature extraction techniques. The results showed that the regression model developed by PLSR based on second derivative (SD) and competitive adaptive reweighted sampling (CARS) resulted in better performance for moisture content. The model showed predictive abilities for moisture content with a coefficient of determination of 0.9075 and a root mean square error (RMSE) of 0.4828 for the training set; and a coefficient of determination of 0.907 and a root mean square error (RMSE) of 0.5267 for the test set; and a relative prediction error of 3.3 and the standard error of 0.307.

## 1. Introduction

Caragana korshinskii pellet feed is rich in nutritional value and can improve the productivity and immunity of ruminants and improve the quality of meat and milk. Caragana korshinskii pellet feed is one of the typical forms of Caragana Korshinskii feed utilization, because the pellet feed has the advantages of easy transportation, reducing dust, reducing feeding loss, and reducing oxidation, etc. [1,2]. In order to make full use of the nutrients in Caragana korshinskii pellet feed, its moisture content needs to be tested. When the moisture content is excessive, it not only increases the growth of bacteria and microorganisms in pelleted feed, but also leads to higher transportation costs; when the moisture content is too low, it makes the hardness greater and reduces the animal feeding rate. Therefore, it is necessary to make a fast and accurate non-destructive determination of the moisture content value of Caragana korshinskii pellet feed.

The measurement of moisture content is often conducted using traditional drying methods, which are associated with issues such as complex procedures and lengthy time consumption. With the development of spectroscopic detection technology, the research of near-infrared spectral equipment for non-destructive testing of feed quality has been increasing. For example, Na Rong et al. [3] conducted a study on alfalfa feed and established a near-infrared spectral prediction model for six conventional nutrients, and the prediction model coefficient of determination were above 0.9. Wang Yongsheng et al. [4] used near-infrared spectroscopy to assess the moisture content and crude protein in sorghum feed and established prediction models that could accurately assess the crude protein and moisture content. However, near-infrared spectroscopy is susceptible to interference from the external environment, has low resolution, and is not sufficiently rich in spatial information [5,6]. Hyperspectral imaging techniques can provide the average spectral information of multiple pixels within an image with high resolution, is not easily disturbed by the external environment, and has the advantages of non-destructive, non-pollution speedy results [7], and is widely used in feed quality inspection research. Jue Zhang et al. [8] predicted the moisture content of silage by an improved discrete particle swarm algorithm and established a partial least squares regression (PLSR) model with a prediction set coefficient of determination of 0.86. Rui Gao et al. [9] evaluated the crude protein content of forage based on hyperspectral imaging and established a PLSR prediction model with a prediction set coefficient of determination of 0.933. Rabanera J D et al. [10] used hyperspectral imaging rapid nondestructive measurement of peanut kernel moisture content using hyperspectral imaging technique and showed a prediction set coefficient of determination of 0.9445. In summary, most of the tests were performed for feed ingredients and less research was performed on finished pelleted feed quality testing.

In order to realize the rapid and nondestructive detection of the moisture content of Caragana korshinskii pellet feed, this study was conducted to rapidly detect the moisture content of Caragana korshinskii pellet feed with the help of hyperspectral imaging technology, to establish a quantitative analysis prediction model of the moisture content of Caragana korshinskii pellet feed by chemometric method, and to validate the model in order to provide a new method for the accurate and rapid detection of the moisture content of Caragana korshinskii pellet feed.

## 2. Materials and Methods

### 2.1. Experimental Materials

**Figure 1:**
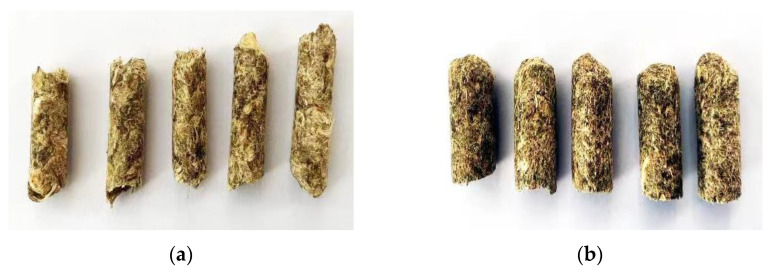
Caragana korshinskii pellet. (**a**) 5 mm particle size; (**b**) 9 mm particle size.

This experiment was conducted with Caragana korshinskii pellet feed from the following sources:

(a) In August 2022, the Caragana korshinskii powder was purchased after crushed and dried at Yuan Shang Grass Industry Company, Hohhot City and Linge County, Inner Mongolia Autonomous Region, where the raw materials came from Hohhot City and Linge County, Inner Mongolia Autonomous Region, and the Caragana korshinskii pellet feed with 6 mm particle size was obtained by granulating using Zhengchang SZLH558 ring pattern pellet mill, as shown in Figure 1a, which was randomly selected and weighed. The individual samples weighed about 140 g, 72 samples in total, numbered into 14 cm × 20 cm self-sealing bags. The samples were stored in the refrigerator (temperature about 8 °C).

(b) In March 2023, the Caragana korshinskii pellet feed with 9 mm particle size purchased from Mengchuanuo feed plant in Baotou, Inner Mongolia Autonomous Region, as shown in Figure 1b, and the raw material came from Ulanqab, Inner Mongolia Autonomous Region. The purchased feeds were randomly selected and weighed, and the individual samples weighed about 140 g. A total of 72 samples were packed into 14 cm × 20 cm self-sealing bags with numbers and stored in the refrigerator (temperature about 8 °C).

**Figure 2:**
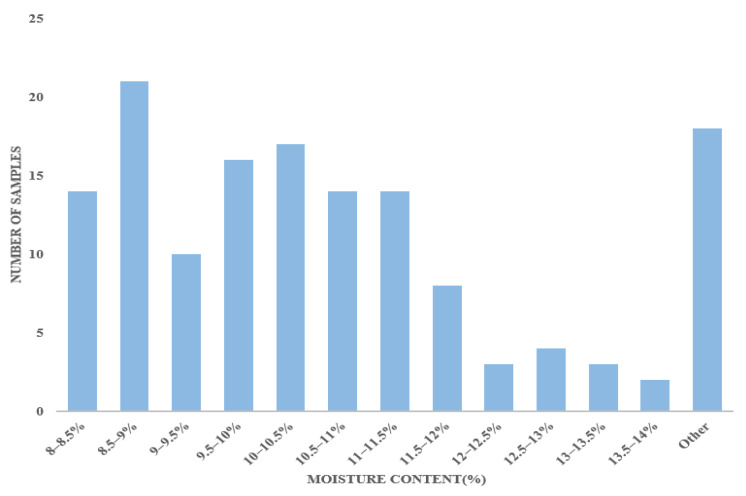
Moisture Content Histogram.

Firstly, the moisture content of Caragana korshinskii pellet feed from different regions was controlled by using vacuum bags to moisturize the feed, and the moisture content was maintained by increasing the moisture content using a water spray bottle to spray water evenly and using vacuum bags to rest for 24 h. The overall moisture content should be below 14%; “Other” refers to feeds with a moisture content outside the range of 8–14%, in order to increase the range of concentration variation in the index to be tested. About 14 g of each sample was randomly selected for the drying test to detect the current moisture content. According to the national standard GB/T 6435-2014 [11] in the electric constant temperature blast drying (DHG-9140A, Shanzhi Instrument Equipment LTD. Shanghai, China) oven set to 105 °C. The weighed feed samples were put into the drying oven, dried continuously for 10 h, and then removed to record the weight, and then put into the drying oven again for 1 h and then removed until the weight change before and after was within 0.001 g. The 144 samples moisture contents were then used to determine the standard error of the laboratory (SEL), which was determined to be 0.153. The number of samples in each moisture content range was expressed as a histogram, as shown in Figure 2.

### 2.2. Test Method

#### 2.2.1. Determination of Moisture Content

Determination of moisture content: Moisture content is determined by using DHG-9140A type electric constant temperature blast drying oven with reference to GB/T 6435-2014 drying method, and the determination formula is as follows:

(1) $$H=\frac{M_{1}-M_{2}}{M_{1}}$$

where H—moisture content; $M_{1}$—mass before drying; and $M_{2}$—mass after drying.

#### 2.2.2. Hyperspectral Data Acquisition

**Figure 3:**
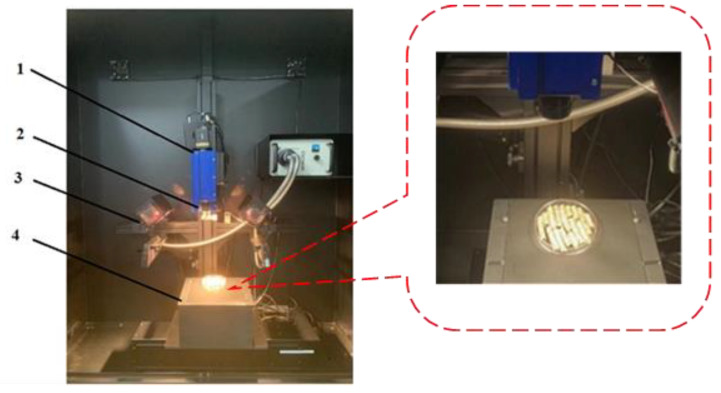
Hyperspectral image acquisition system. 1. Spectrometer. 2. Lens. 3. Halogen lamp. 4. Mobile displacement platform.

The hyperspectral imaging system of ISUZU OPTICS LTD (Taiwan, China) was used in this experiment, which mainly consists of an imaging spectrometer (ImSpector N25E, SPECTRAL IMAGING LTD, Oulu, Finland), a fiber optic halogen lamp (model 3900-ER, 21 V/150W, Advanced illumination, Inc, Rochester, VT, USA), a mobile displacement platform (IRCP0076-1, COM, Taiwan, China), a computer with data acquisition software, a dark box to prevent the influence of interfering light, and lenses. The schematic diagram of the hyperspectral imaging system for collecting Caragana korshinskii pellet feed is shown in Figure 3. The hyperspectral imaging device was preheated for 30 min before data collection. The lens location was changed to be able to catch the entire Petri dish where the sample was placed, and the light source was adjusted to maximize the energy value of the light source. The instrument parameters during the acquisition process were set as follows: the acquisition range of the hyperspectral imaging system was 935.5~2539 nm, the exposure time was 1.94 ms, the spectral resolution was 6.3 nm, the moving displacement platform range was 100~280 mm, and the speed was 21.25 mm/s.

#### 2.2.3. Spectral Image Black and White Correction

**Figure 4:**
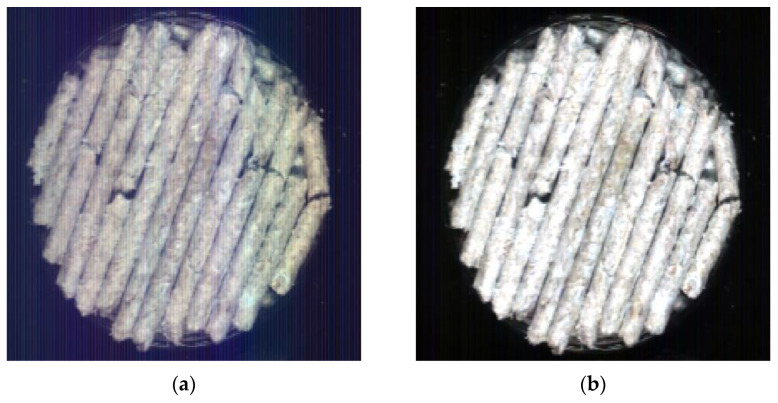
Black and white correction. (**a**) Pre-correction; (**b**) Post-correction.

In order to capture high quality image data, the hyperspectral imaging system should be set up in advance and the original spectral image ($I_{0}$) of the collected Caragana korshinskii pellet feed samples should be corrected in black and white. The blackboard and whiteboard correction can reduce the problem of high noise due to uneven distribution of light intensity at each wavelength, and also ensure that the images collected by the spectroscopy system can be converted to reflectance spectra more completely [12]. First, under the same system conditions as the sample acquisition, the camera was covered with a lens cap and scanned to obtain the calibration image information in all-black ($I_{B}$). Second, a standard white calibration plate with 99% reflectance was scanned to obtain the calibration image information in all-white ($I_{W}$). Finally, the calibration was performed by Equation (1), as shown in Figure 4.

(2) $$I=\frac{I_{0}-I_{B}}{I_{W}-I_{B}}$$

where: $I_{0}$—the original spectral image; $I_{B}$—the all-black calibration image. $I_{W}$—all-white calibration image; and I—corrected hyperspectral image.

### 2.3. Data Processing Methods

#### 2.3.1. Extraction of Average Spectral Data

By using python, the cv2 library was used to select the region with Caragana korshinskii pellet feed in the hyperspectral image, the gdal module in the osgeo library was used to extract the spectral data of all pixels in the selected region of the hyperspectral image, and the spectral data of all pixel points were averaged as the original spectral data 935.5~2539 nm (256 bands in total). The extracted raw spectra were processed for the spectral data in the range of 960~2489 nm (244 bands in total) after eliminating the first and last ends with low signal-to-noise ratio.

#### 2.3.2. Abnormal Sample Rejection

In non-destructive testing based on spectroscopic techniques, anomalous samples can affect the performance of the model and the prediction accuracy. Therefore, Monte Carlo cross validation (MCCV), which can effectively detect the abnormal values in the direction of spectral and property arrays and has a higher ability to identify the anomalous samples compared with the traditional methods, was used to reject the anomalous samples.

#### 2.3.3. Preprocessing Methods

When imaging hyperspectral imaging systems, the data are often affected by the instrument background, uneven particle distribution or different particle sizes, and instrument signal noise. In order to improve the prediction accuracy and stability of the model it was necessary to pre-process the collected data to remove the interference factors. The preprocessing effects can be divided into four categories: scattering correction, baseline correction, smoothing processing, and scale scaling [13,14,15]. Due to the variability of instrument errors and environmental factors, there is not yet a universal spectral preprocessing algorithm with high applicability, and no accepted evaluation parameters exist. The preprocessing methods used in this study are mainly first derivative (FD), second derivative (SD), multiplicative scattering correction (MSC), standard normal variate (SNV), SG (Savitzky-Golay) convolution smoothing (The derivative order is 2 and the smoothing point is 9), mean center (MC), and MinMax normalization (MMN). A PLSR model is developed for the pre-processed spectral data to determine the optimal pre-processing method.

#### 2.3.4. Model Building and Evaluation

The partial least squares regression (PLSR) and random forest regression (RFR) algorithms were used to develop a quantitative spectral analysis model for the moisture content of Caragana korshinskii pellet feed. The performance of the models was evaluated mainly by the coefficient of determination ($R^{2}$) and root mean square error ($R_{MSE}$), and relative prediction error (ratio of performance to predictive deviation, RPD) between the sample reference value and the predicted value. Among them, the closer the $R_{C}^{2}$ and $R_{MSEC}$ of the model are to 1 and 0, the better the modeling quality is; the closer the $R_{P}^{2}$ and $R_{MSEP}$ are to 1 and 0, the better the model prediction ability is; and the closer $R_{\mathrm{CV}}^{2}$ is to 1 and the closer $R_{MSECV}$ is to 0, the better the model performance is. Large values of $R_{C}^{2}$ and $R_{P}^{2}$ with less difference and small values of $R_{MSEC}$ and $R_{MSEP}$ with less difference show that the model accuracy and stability is, and the model performance is better when the RPD value is greater than 3. The maximum $R^{2}$ value and the minimum value of standard error (SE) indicate the ability of the model to represent a good relation between dependent and independent variables. The above data processing procedures were implemented on MATLAB2018b software.

#### 2.3.5. Competitive Adaptive Reweighted Sampling (CARS) Method

The CARS algorithm is a feature wavelength selection method based on Monte Carlo sampling and partial least squares (PLS) model regression coefficients [16,17], and can build a PLS model by continuously selecting a subset of wavelength combination variables and determining the optimal subset by selecting the smallest root mean square error of cross-validation, which corresponds to the selected of characteristic wavelengths, thus determining the optimal combination of variables. The specific algorithm is analyzed as follows:

(1) A certain percentage of samples are extracted using Monte Carlo sampling method to establish the PLS model.

(2) The absolute value of the regression coefficients of each variable in the PLS model is calculate, *B* [18]; the weight value of each regression coefficient, $w_{i}$ is calculated based on *B*

(3) $$w_{i}=\frac{B_{i}}{\sum_{i=1}^{m}B_{i}}$$

The larger the $w_{i}$ value, the more important the variable is.

(3) Exponentially decreasing function (EDF) is used to remove the variables with small absolute values of regression coefficients from the PLS model. The residual rates of spectral variables $r_{i}$ are:

(4) $$r_{i}=\mu e^{-ki}$$

Among them, $\mu ={\left(\frac{n}{2}\right)}^{\frac{1}{N-1}}$, $k=\frac{\ln\frac{n}{2}}{N-1}$.

(4) After repeating N times of sampling, the $R_{MSECV}$ corresponding to N PLS models was obtained, and the smallest one was selected as the optimal subset of variables.

#### 2.3.6. Successive Projections Algorithms (SPA)

The SPA algorithm extracts the main information of the spectral data mainly by gradually selecting the important projection directions in the spectral data [19]. The partial least squares (PLS) model is established by taking the selected feature bands and the content of each parameter. The optimal feature wavelength extraction method is selected based on the model results. The SPA advantage is in extracting the spectral variables containing the least redundant information with the lowest covariance by a simple projection operation [20]. The specific implementation steps are as follows:

(1) Arbitrarily select a column j in the spectral matrix and assign column j of the modeled set to $x_{j}$, denoted as $x_{k(0)}$;
(2) Denote the set of remaining vector positions as S;

(5) $$S=\{j,1\leq j\leq J,j\notin \left\{k\left(0\right),\;\ldots ,k\left(n-1\right)\right\}\}$$

(3) Compute the projections of $x_{j}$ onto the remaining column vectors separately:

(6) $$P_{\mathrm{xj}}={\;x}_{j}-\left(x_{j}^{T}x_{k\left(n-1\right)}\right){\left(x_{k\left(n-1\right)}^{T}x_{k\left(n-1\right)}\right)}^{-1},j\in S$$

(4) Denote as

(7) $$k\left(n\right)=argmax(∥P_{xj}∥,j\in S)$$

(5) Notate

(8) $$x_{j}*P_{xj},j\in S$$

(6) Let n = n + 1, if n < N repeat Equation (5)

where J is the spectral data; $x_{k\left(0\right)}$ is the initial iteration vector; n is the number of samples; P is the number of spectral wavelengths; N extracts the number of characteristic wavelengths; $P_{xj}$ is the vector projection; S is the set of remaining vector positions; and k(n) is the maximum projected vector for wavelengths.

## 3. Results

### 3.1. Average Spectral Data Extraction

**Figure 5:**
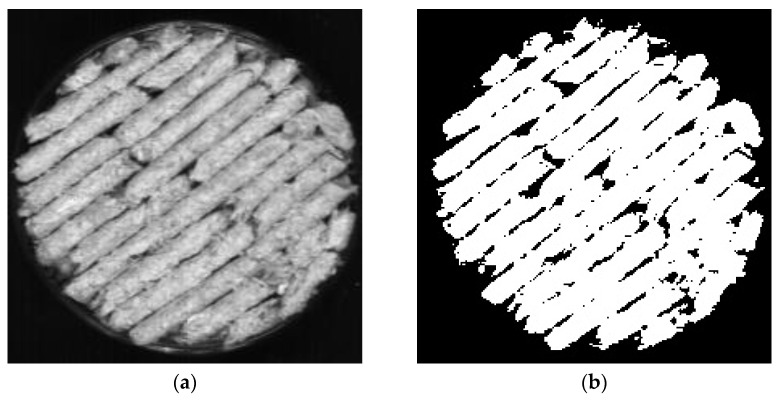
Selection of Caragana korshinskii Pellet Feed Area. (**a**) Original spectrogram; (**b**) Algorithm selected region.

In order to select the spectral data with obvious characteristics and representativeness, the spectral data in the Caragana korshinskii pellet feed were extracted using python. Unlike the traditional manual selection of regions to extract the average spectrum, the algorithm can automatically extract the spectral data for the entire spectral image where samples exist, while averaging the spectral data of all pixel points as the original spectral data, which greatly reduces the workload of manually selecting regions. The extracted regions are shown in Figure 5. Figure 5a shows the original spectral image taken by the hyperspectral imaging system, and b shows the part of the white area where the Caragana korshinskii pellet feed sample exists selected by python. It can be seen from Figure 5 that the area is more comprehensively selected by the algorithm, and the area where the pellet feed does not exist can be identified, which not only ensures the accuracy of identification, but also reduces the workload.

### 3.2. Anomalous Sample Rejection

**Figure 6:**
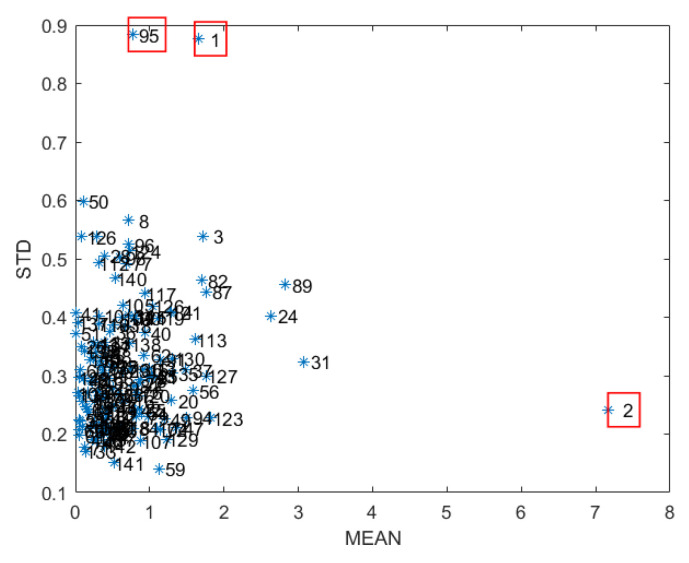
Mean variance distribution of moisture content. (‘*’ indicates the Nth sample; red boxes represent outliers to be eliminated).

The method mainly takes 75% of the samples as the correction set by Monte Carlo random sampling (MCS) to build the PLS regression model. The remaining part is used as the prediction set with 1000 cycles to obtain a set of prediction residuals for each sample, find the mean value (MEAN) and variance (STD) of the prediction residuals for each sample, and make MEAN-STD Figure 6 [21]. The PLSR models were developed for the moisture content before and after sample removal, respectively, to compare the accuracy of the models as well as the prediction accuracy. From Figure 6, it can be seen that certain samples obviously deviate from the main sample, such as No. 1, No. 2, and No. 95 in the figure, and these samples can be considered as anomalous samples and will be rejected.

By building the PLSR model before and after culling, it can be established that the model accuracy is improved from the coefficient of determination $R_{C}^{2}=0.7708$, $R_{P}^{2}=0.6697$, and the root-mean-square error $R_{MSEC\;}=0.83813$, and $R_{MSEP}=0.95976$ before the culling, to the coefficient of determination $R_{C}^{2}=0.8364$, $R_{P}^{2}=0.8186$, and root-mean-square error $R_{MSEC}=0.62132$, $R_{MSEP}=0.68395$. The prediction accuracy of the model was improved, which is consistent with the studies of two scholars [22,23], in which the accuracy of the established regression prediction model was improved after removing the abnormal samples by using MCCV. This indicates that there are anomalous data in the measured moisture content and the extracted spectral information, which may be due to human factors or instrumental errors in the measurement of moisture content, resulting in anomalous results in some measurements. Furthermore, it is also possible that when taking the spectra of some samples, they were interfered with by the environmental factors or the preparation of the samples, which resulted in the influence of the optical properties of the samples.

### 3.3. Spectral Data Pre-Processing

**Figure 7:**
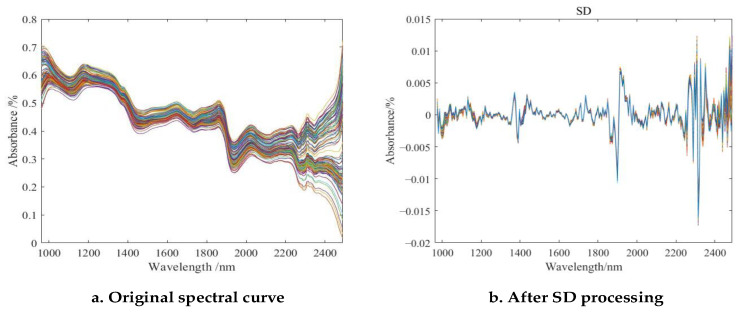
Original spectral curve of Caragana korshinskii pellet feed and spectrum after SD pretreatment. (Different color curves represent different samples).

After processing the spectral data by the seven preprocessing methods, the PLSR models were built separately, and the preprocessing results are shown in Figure 7b and Table 1. As shown in Figure 7a, there are several absorption peaks in the whole band, among which the absorption peaks are obvious in the ranges of 1093.4~1118.7 nm, 1450~1484.4 nm, 1843~1868.2 nm, and 1918.4~1943.6 nm. The absorption peaks near 1093.4~1118.7 nm may be related to the protein and free amino acids in the feed, the absorption peaks near 1450 nm may be related to the C-H bond or the O-H doublet (water) of the fat in the feed. The absorption peaks near 1843~1868.2 nm may be related to the carboxylic acid (-COOH) and C=O doublet contained in the feed or water molecules, and those near 1918.4~1943.6 nm may be related to the C-H bonds of fats in the feed [24,25].

From Table 1, the optimal pretreatment method was screened using the PLSR and RFR models, and the best full-band model was obtained using the SD method in building the prediction model for moisture content, where the coefficient of determination $R_{C}^{2}=0.8916$, $R_{P}^{2}=0.8503$, root mean square error $R_{MSEC}=0.5137$, and $R_{MSEP}=0.5953$, which indicates that the absorption peaks of the samples. This indicates that the absorption peaks of the samples are more concentrated and have a more obvious change in shape with respect to the baseline. As shown in Figure 7, the spectra of the Caragana korshinskii pellet feed after the second-order derivative treatment, which enhanced the height and shape of the absorption peaks of the samples, removed the influence of the baseline, and made the differences among the samples more prominent [26]. Therefore, this paper will be analyzed based on the data after SD preprocessing.

### 3.4. Feature Wavelength Extraction

The volume of hyperspectral imaging data is large and wavelength is even larger, so there may be some irrelevant features which will not play any role in the algorithm. Additionally, there will also be some redundant features, which can be introduced by other features or will not add any information; the purpose of feature wavelength selection is to leave the relevant features and remove the irrelevant and redundant features. Since these redundant and irrelevant features exist, they increase the modeling time and model complexity, and reduce the stability and prediction accuracy of the model. Therefore, feature wavelength selection is needed to improve model prediction accuracy and simplify the model. In this study, competitive adaptive reweighted sampling (CARS) and successive projections algorithms (SPA) were used to extract the feature wavelengths from the spectral data after pretreatment of Caragana korshinskii pellet feed.

#### 3.4.1. Competitive Adaptive Reweighted Sampling (CARS) Method

**Figure 8:**
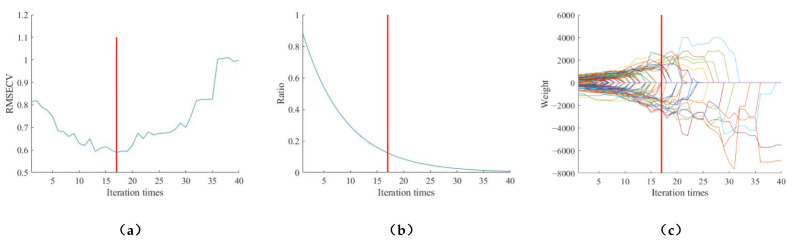
SD-CARS screening results for moisture content. (Different color curves represent different variables) As the number of samples increases (**a**) Trends in RMSECV values; (**b**) Trends in the percentage of extracted variables; (**c**) Trends in regression coefficients for each variable.

The characteristic wavelengths were extracted from the moisture content SD processed spectra using CARS by setting the number of Monte Carlo samples to 40 and using the 10-fold cross-validation method. Figure 8a shows a graphical representation of the exponentially decreasing function, in the first stage, the number of wavelengths decreases rapidly, and in the second stage, the number of wavelengths decreases very slowly to achieve fine selection. Figure 8b shows the variation in the root mean square error of the cross-validation with the increase in sampling times. In the first 17 sampling times, the error keeps decreasing with the increase in sampling times, which indicates that the spectral data that have less correlation with moisture content are being eliminated, and after the 17 sampling times, the error increases with the increase in sampling times, which indicates that the spectral data with high correlation with moisture content are also being eliminated due to the high selectivity. Figure 8c shows the change in the regression coefficient path with the increase in the sampling times, and the corresponding number of sampling times is 17 when the root mean square error of the cross-validation is smallest. There were 34 characteristic wavelengths obtained after the 17th sampling, in the order of 1030.28, 1036.59, 1087.11, 1112.37, 1137.62, 1143.93, 1232.27 1238.57, 1364.69, 1370.99, 1377.29, 1408.8, 1478.1, 1490.7, 1497, 1673.25, 1692.12, 1717.29, 1729.86, 1736.15, 1793.74, 1868.17, 1905.87, 1962.4, 2087.96, 2100.51, 2131.88, 2156.97, 2163.25, 2194.61, 2269.85, 2294.92, 2345.05, and 2370.11 nm, which accounted for 13.9% of the full spectral band.

#### 3.4.2. Successive Projections Algorithms (SPA)

**Figure 9:**
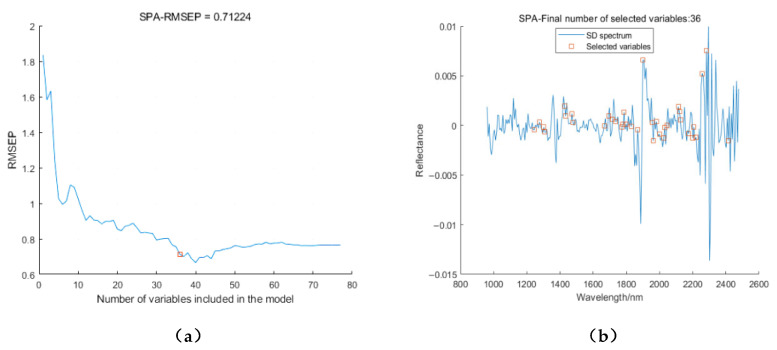
SPA feature extraction results of moisture content. (**a**) Trends in RMSE as the number of variables increases; (**b**) Location of selected variables.

Using SPA to extract the characteristic wavelengths of the moisture content after SD treatment of the spectrum; as shown in Figure 9a, when the number of variables is 36, the minimum RMSE is 0.71224. Figure 9b shows the positions of the selected characteristic wavelengths, in order, 1244.9, 1276.4, 1301.6, 1307.9, 1427.7, 1434.0, 1471.8, 1478.1, 1667.0, 1692.1, 1717.3, 1736.2, 1773.9, 1780.2, 1786.5, 1799.0, 1830.5, 1868.2, 1899.6, 1956.1, 1962.4, 1981.2, 2000.1, 2025.2, 2031.5, 2044.0, 2113.1, 2119.3, 2125.6, 2175.8, 2200.9, 2207.1, 2219.7, 2257.3, 2382.6, 2414.0 nm accounted for 14.7% of the full spectral band.

### 3.5. Establishment of Predictive Model and Distribution Map for Moisture Content of Caragana Korshinskii Pellet Feed

After the spectral data of Caragana korshinskii pellet feed were pre-processed by SD, the characteristic wavelengths were extracted by CARS and SPA, and the PLS and RFR models of the full band and characteristic wavelengths of moisture content of Caragana korshinskii pellet feed were established.

#### 3.5.1. Establishment of PLSR Model

The partial least squares regression (PLSR) algorithm is a relatively common modeling method in chemometric analysis, which combines the advantages of several algorithms and is suitable when the number of variables is large, the samples are small, and there are multiple correlations [27,28]. The algorithm can better deal with data covariance by considering both spectral data and chemometric values on modeling in the process of calculation. By using 10-fold cross-validation during the process of cross-validation, different numbers of principal components (referred to as “Number of components”, nc) can be tested starting from 1 and gradually increased until reaching the maximum value of nc. The root mean square error can then be compared for different numbers of principal components to determine the optimal number of latent variables and thus achieve the best predictive performance.

#### 3.5.2. RFR Model Building

The random forest regression (RFR) algorithm is an integrated learning model based on decision trees. It constructs multiple decision trees by randomly selecting samples and features in the training set and combining them into an integrated model. In prediction, the random forest model averages (or averages by weight) the results of each decision tree to obtain the final prediction results [29]. The RFR model has good generalization and adaptability, can effectively solve the nonlinear problems in modeling, and has good fault tolerance for outliers and noise. The RFR model needs to specify the number of trees as well as the number of leaf nodes. In order to find out the optimal number of trees and the number of leaf nodes, the test was performed starting from the default value of 100 for the tree and tested to 1000 in intervals of 100 with the number of leaf nodes started from the default value of 1 and tested to 10 at intervals of 1. Finally, the number of trees was determined to be 100 and the number of leaf nodes was determined to be 5.

The results of different combinations of models are shown in Table 2. From the analysis of the number of feature wavelengths Table 2, it can be seen that under the same algorithm with different indicators, the number of feature wavelengths extracted by using CARS algorithm is less compared with that extracted by using the SPA algorithm, and in the process of moisture content model building, the prediction model built by CARS is better compared with that built by SPA, probably because the SPA algorithm extracts the noise when screening the feature variables or the wavelengths with higher correlation with feed moisture content are removed. It can also be seen from Table 2 that the PLSR model has a better prediction effect than the RFR model, in which the SD-SPA-RFR model has the lowest prediction accuracy with a coefficient of determination of 0.8243 and root mean square error of 0.6303; the SD-CARS-PLSR is the optimal combination with a coefficient of determination of 0.9075 and root mean square error of 0.4828. By comparing the prediction models at full wavelength and feature wavelength, we can see that the prediction accuracy of SD-CARS-PLSR improves by 0.0567 and the root mean square error of the test set decreases by 0.0686 compared with that of SD-PLSR, which indicates that the extraction of feature wavelength can improve the prediction performance of the model. The SE is considered a statistical parameter for assessing the predictive ability of a model and, as a rule of thumb, a model with an SE that is less than twice the SEL is considered excellent [30]. Analyzing the magnitudes of the SE values in Table 2, it can be observed that the optimal predictive model has an SE of 0.307 and SEL of 0.153. The SE value is nearly twice the SEL value, indicating that the model performs well.

**Figure 10:**
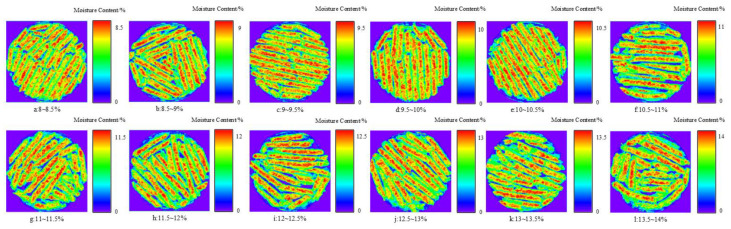
Distribution of moisture content at different ranges. (Different colors in the graph indicate different moisture content, red represents the maximum moisture content value and blue represents the minimum moisture content value.)

In hyperspectral imaging, each pixel corresponds to a spectral curve, and the moisture content of each pixel in the sample can be calculated. To calculate the moisture content of the entire sample, all pixels in the hyperspectral image need to be processed. In this study, the regression coefficient equation of the optimal regression model was used to estimate the moisture content of each pixel on the hyperspectral image of Caragana korshinskii pellet feed. The resulting image displays different colors corresponding to different levels of moisture content. As shown in Figure 10, the distribution map of the sample under different moisture content ranges indicates that the control of feed moisture content was relatively uniform, with no uneven distribution of moisture content in the samples. Additionally, in the distribution of moisture content on individual sorghum particles, it was observed that the moisture content was lower at the edges of the particles, possibly due to environmental factors during hyperspectral imaging. By visualizing the distribution of moisture content in Caragana korshinskii pellet feed, we can intuitively observe the moisture content in different areas, which can help us analyze the internal variations in the feed.

## 4. Discussion

In this study, we propose and develop a fast and non-destructive model which is capable of measuring moisture content. The performance of our proposed SD-CARS-PLSR model is mainly evaluated by R2, RMSE, and RPD metrics. On the training set, the R2 is 0.9075 and the RMSE is 0.4828, which indicates that the model is able to accurately fit the training data; on the test set, the R2 is 0.907 and the RMSE is 0.5267, which suggests that the model is able to have good prediction ability even on unseen data. In addition, the root mean square error on the test set is slightly higher than that on the training set, which may be related to the fact that the distribution of the sample features in the test set differs from that of the training set. The RPD value of 3.3 indicates that the model has high prediction accuracy and has the potential for practical application.

In previous studies, for molded pellet feed, spectral detection was mostly performed after crushing [31,32]. In order to eliminate the tedious step of crushing, this study adopted the method of direct detection of molded pellets, but in the process of extracting the average spectral data problems such as heavy workload, insufficient extraction, and inaccurate data arose. Therefore, this study proposes a method for extracting average spectral information for pellet feed.

However, there are some shortcomings and areas for improvement in the study. Firstly, only two regions of Caragana korshinskii pellet feed were used as the research object in this study; secondly, there may have been some errors during the sample collection process which resulted in the model’s accuracy not being very high. Therefore, more samples and optimization of the model can be considered to improve the accuracy of the model in subsequent studies.

## 5. Conclusions

Using Caragana korshinskii pellet feed as the research object, the average spectra of Caragana korshinskii pellet feed samples in the range of 935.5~2539 nm were collected by hyperspectral imaging technology, and the moisture content of the feed was measured by an electric thermostatic blast dryer. Then, a prediction model of hyperspectral and feed moisture content was established, which concluded that hyperspectral imaging technology could achieve the prediction of moisture content of Caragana korshinskii pellet feed. The main conclusions are as follows:

(1) For fast and accurate detection of moisture content of Caragana korshinskii pellet feed, the average spectral information of Caragana korshinskii pellet feed should be extracted by means of python language algorithm.

(2) To achieve accurate detection of moisture content of Caragana korshinskii pellet feed, the extracted spectral data and the measured moisture content are rejected by the MCCV method. Seven pre-processing methods were applied to the data after the rejection of abnormal samples, and it was found that the model results were better after processing by the SD method according to the establishment of the pre-processed full-band PLSR model. For the spectral data after SD preprocessing, the characteristic wavelengths were selected using CARS and SPA methods, and the characteristic wavelengths with high correlation with moisture content were obtained using two characteristic wavelength selection methods, accounting for 13.9% and 14.7% of the full spectrum, respectively.

(3) Based on the above preprocessing method and feature wavelength selection method, the relationship models of full spectrum and feature wavelengths of moisture content were established using PLSR and RFR, respectively. The results show that the established characteristic wavelength prediction model has a faster calculation speed and higher prediction accuracy compared with the full-band prediction model. Among the established moisture content prediction models, SD-CARS-PLSR was the optimal combination with $R_{C\;}^{2}=0.9075$, $R_{MSEC}=0.4828$; $R_{P}^{2}=0.907$, $R_{MSEP}=0.5267$; $R_{CV}^{2}=0.8628$, and $R_{MSECV}=0.5618$. In summary, hyperspectral technology can achieve nondestructive and rapid detection of the moisture content in Caragana korshinskii pellet feed in the state of densely formed pellets.

## Figures and Tables

**Table 1:** Prediction results of PLSR And RFR modeling using different preprocessing methods.

Model	Pre-Processing	Number of Components	Cross-Validation		Training Set		Test Set	
			$R_{MSECV}$	$R_{CV}^{2}$	$R_{MSEC}$	$R_{C}^{2}$	$R_{MSEP}$	$R_{P}^{2}$
PLSR	RAW DATA	15	0.7253	0.7857	0.6213	0.8364	0.6840	0.8186
	SNV	15	0.7525	0.7747	0.6281	0.8115	0.7493	0.8100
	MSC	12	0.7296	0.7736	0.6539	0.8053	0.8411	0.7864
	SG	15	0.7550	0.7878	0.5918	0.844	0.6043	0.8266
	FD	11	0.7594	0.7900	0.5510	0.8677	0.6151	0.8458
	SD	10	0.7077	0.7719	0.5137	0.8916	0.5953	0.8503
	MMN	13	0.7218	0.7982	0.6280	0.8098	0.7676	0.7557
	MC	13	0.7581	0.7857	0.6732	0.8023	0.6538	0.802
RFR	RAW DATA		0.7940	0.7560	0.5692	0.8843	0.7283	0.7457
	SNV		0.8326	0.7666	0.5058	0.8939	0.7845	0.8077
	MSC		0.8100	0.7760	0.5146	0.895	0.7311	0.8151
	SG		0.9030	0.6979	0.8557	0.8633	0.8557	0.7689
	FD		0.7872	0.7704	0.4691	0.9136	0.6538	0.8483
	SD		0.7447	0.7905	0.4725	0.9177	0.5532	0.8685
	MMN		0.8670	0.7460	0.5054	0.8960	0.7663	0.8086
	MC		0.8654	0.7136	0.5787	0.8860	0.6164	0.7795

**Table 2:** Results of PLSR and RFR detection models for moisture content of Caragana korshinskii pellet feed established by different variable selection methods.

Models	Algorithm Combinations	nc	Characteris-Tic Variables	Cross-Validation		Training Set		Test Set		RPD	SE
				$R_{MSECV}$	$R_{CV}^{2}$	$R_{MSEC}$	$R_{C}^{2}$	$R_{MSEP}$	$R_{P}^{2}$		
PLSR	SD-CARS-PLSR	9	34	0.5618	0.8628	0.4828	0.9075	0.5267	0.907	3.3	0.307
	SD-SPA-PLSR	10	36	0.7142	0.7953	0.5486	0.8664	0.6287	0.8653	3.2	0.313
RFR	SD-CARS-RFR		34	0.7173	0.8044	0.4946	0.9025	0.6991	0.8324	2.4	0.471
	SD-SPA-RFR		36	0.7288	0.7878	0.5116	0.9038	0.6303	0.8243	2.3	0.495

## Data Availability

Not applicable.
